# Effects of the COVID-19 pandemic on youth mental health: a cross-sectional study on eating disorder patients and their unaffected siblings

**DOI:** 10.1186/s13034-023-00698-5

**Published:** 2024-01-06

**Authors:** Paolo Meneguzzo, Alessio Ajello, Mauro Domenico Consolati, Enrico Ceccato, Antonio Vita, Alessandra Sala, Paolo Santonastaso

**Affiliations:** 1 Department of Neuroscience, University of Padova, Via Giustiniani 2, 35128 Padua, Italy; 2 Padova Neuroscience Center, University of Padova, Padua, Italy; 3 Department of Clinical and Experimental Sciences, University of Brescia, Brescia, Italy; 4 Department of Mental Health and Addiction Services, ASST Spedali Civili of Brescia, Brescia, Italy; 5 Mental Health Department, Vicenza Eating Disorders Center, Azienda ULSS8 “Berica”, Vicenza, Italy

**Keywords:** Adolescence, Eating disorder, Posttraumatic, COVID-19, Siblings, Psychopathology

## Abstract

**Background:**

Adolescence has emerged as a particularly vulnerable phase during the COVID-19 pandemic, with eating disorders (EDs) representing a prominent psychopathological challenge linked to the restrictions imposed by the pandemic. Emerging evidence suggests that not only individuals with EDs but also their healthy siblings (HS) may experience unique psychological effects in this context. However, the existing literature on this topic remains limited. This study seeks to examine and compare the effects of the pandemic on adolescents and adults, with a specific focus on the impact of containment measures, disruptions in daily routines, and alterations in life trajectories, for both individuals with EDs and their HS.

**Methods:**

We enrolled 273 individuals, including those diagnosed with EDs and their HS. Among the participants, 120 were under the age of 19. Multiple self-report questionnaires were administered to assess the psychological impact of 1 year of the COVID-19 pandemic. These assessments covered a range of psychological constructs, including posttraumatic symptoms, general psychopathology, and eating-related concerns.

**Results:**

Notably, adolescent patients with EDs demonstrated the highest psychopathological scores within our sample. They were the sole subgroup to surpass clinical cutoffs, exhibiting more pronounced issues concerning eating-related concerns and general psychological well-being. Our findings also shed light on the unique experiences of HS during the pandemic.

**Conclusion:**

Our findings highlight the specific psychological burden endured by adolescents with EDs throughout the COVID-19 pandemic, emphasizing the vulnerability of this demographic. Moreover, the experiences of HS, often overlooked in the literature, warrant increased attention in future health programs and interventions.

## Background

Adolescence represents a distinct developmental stage characterized by a significant reliance on peer connections for emotional support and social development [1]. It is a period marked by vulnerability due to various transitions [2], and the COVID-19 crisis and associated lockdowns have introduced additional challenges for adolescents, including social distancing, isolation, and disrupted routines. These disruptions have had multiple consequences on adolescents’ lives, including increased stress, concerns for their families, abrupt interruptions in education and sports activities, and heightened exposure to social media [3].

These factors have contributed to a rise in psychological distress among young people, leading to increased demand for psychological and psychiatric support, particularly for depression, anxiety, and posttraumatic stress [4, 5]. Notably, adolescents have faced unique challenges during the pandemic, such as eating and weight concerns [6], with differences between ages in the general population [7]. Emerging data reveal that some adolescents resorted to restrictive and compensatory behaviors to lose weight during the pandemic, resulting in an uptick in referrals to child and adolescent eating disorders services [8, 9]. Moreover, adolescents’ heightened reliance on social media also exposed them to body image insecurities, exacerbating eating disorder behaviors [10]. Examining the potential effects of the pandemic, longitudinal data reveal a specific association between experiencing increased conflict at home, sensation seeking, and lack of perseverance impulsivity in the development of eating psychopathology [11], showing the need for specific evaluations.

Even before the pandemic, eating disorders were prevalent among adolescents, with a higher incidence in females, severely impacting their quality of life [12, 13]. The environments in which adolescents live can influence the development of eating disorders, with factors like social pressure for the ‘perfect’ body, food availability, competitiveness, identity formation, relationship development, and emotion regulation all contributing as potential risk factors [14–18]. The disruption of these factors during the pandemic, akin to traumatic events, has been identified as a likely cause of symptom deterioration [19, 20]. This element should be taken into consideration due to the worse psychological burden reported by individuals with ED who have a history of trauma [21] and the existing theory of an ecophenotype subgroup of ED patients characterized by worsened psychopathology and their ability to respond to stress [22, 23]. Recently, it has also been proposed to consider the COVID-19 pandemic as a *collective trauma* or *psychotrauma* due to the presence of globally shared emotional connections arising from the discrepancy between the threat and coping abilities [24]. However, these aspects are not definitive, even though the pandemic has shed light on the relevance of environmental factors in EDs [25].

When considering environmental interactions, the family unit should be taken into serious consideration in order to understand what we have encountered over the last few years. Indeed, families have faced increased stress levels due to confinement, changes in service availability, and the perceived responsibility in the treatment pathways of their loved ones [26, 27]. However, few studies have considered caregivers, and even fewer have included unaffected siblings [28–31]. Preliminary data have shown that specific interpersonal sensitivity is a vulnerability in responses to pandemic confinements in both people with EDs and healthy siblings [28], but no specific evaluations were done as regards ages. While siblings share environmental and biological elements that are considered risk factors for EDs [32, 33] and could allow for increased specific knowledge about the effects of the pandemic, examining the specific role of age in these groups could help us understand what has been observed in recent years.

Therefore, this study investigates the impact of COVID-19 lockdowns on individuals with EDs and their siblings, considering the distinct responses of adolescents and adults to prolonged exposure to the pandemic’s challenges. The research addresses the complex interplay between adolescence, the pandemic, and EDs. Our primary hypothesis posits that adolescents, in both clinical and non-clinical groups, will show elevated levels of psychopathology compared to adults, signaling a more profound impact on the well-being of young individuals resulting from the pandemic.

## Methods

### Participants

The study participants were recruited between January 2021 and September 2021 from the local Mental Health Department’s Vicenza Eating Disorders Center and the Mental Health and Addiction Services of Brescia’s Eating Disorders Center. This period has provided an opportunity to evaluate the long-term effects of repeated lockdowns and increasing social distancing measures that characterized Northern Italy in 2021, including remote schooling, mandatory mask-wearing, and limitations on movement between different areas.

A trained psychiatrist used an in-person semi-structured clinical interview for DSM-5 (SCID-5) to evaluate individuals with EDs to fulfill the DSM-5 criteria for an ED. All the possible ED diagnoses have been included in the clinical group. Patients' healthy siblings (HS) were enrolled as a comparison group, if present, and were screened to exclude current or past clinical or subclinical ED symptoms. For each patient, only one sibling who was closest in age was included in the study. All the participants were recruited via direct invitations and were volunteers.

The criteria applied to patients and controls were as follows: All participants were required to be between 14 and 40 years old, aligning with the typical age range of patients treated at the ED unit. In addition, they should have no history of psychotic symptoms or severe medical conditions. HSs were excluded if they had a current or previous clinical or subclinical ED diagnosis or any other psychiatric conditions. The study design was approved by local Ethical Committees (VI-04/2021 and BS-4703/2021) and complied with the Declaration of Helsinki and its later amendments. All the participants—or their parents if they were under the age of 18—signed informed consent forms.

### Measures

A demographic questionnaire was collected from all participants to gather various details, such as age, height, weight, years of education, previous history of COVID-19 infection, and several self-report questionnaires. Self-report questionnaires included were: the Eating Disorder Examination Questionnaire (EDE-Q), the brief Symptom Checklist (SCL-58), the revised version of the Impact of Event Scale (IES-R), and the COVID Isolation Eating Scale (CIES), regarding the effects linked to the pandemic social restrictions. All participants were instructed to focus on pandemic-related experiences when completing the surveys.

The Italian EDE-Q is a 28-item self-report questionnaire focused on eating symptomatology and concerns [34, 35]. It comprises four subscales and a total score, with higher scores indicating greater psychopathology. In the current investigation, Cronbach’s α was 0.96 for the global score, 0.86 for the restraint subscale, 0.85 for the eating concern subscale, 0.92 for the shape concern subscale, and 0.90 for the weight concern subscale.

The SCL-58 is a self-report questionnaire derived from the revised version of the SCL-90. It is primarily used to assess general psychiatric symptomatology and distress [36–38]. It has five subscales and a total score: somatization, obsessive–compulsive, interpersonal sensitivity, depression, and anxiety. Responses are limited to a five-point Likert scale, with higher scores reflecting worse symptomatology than lower scores. In the current investigation, Cronbach’s α was 0.90 for the global severity index, somatization = 0.88, obsessive–compulsive = 0.86, interpersonal sensitivity = 0.84, depression = 0.88, and anxiety = 0.82.

The IES-R is a 22-item self-report questionnaire focused on subjective distress caused by specific life events. This has been the primary questionnaire used in research to evaluate the effects of the COVID-19 pandemic [39, 40]. In our study, participants were asked to think specifically about the COVID-19 pandemic. It comprises a total score and three subscales: intrusion, avoidance, and hyperarousal. The clinical relevance cutoff for the impact of an event is 24. A score of 33 or above represents a possible diagnosis of posttraumatic stress disorder [41]. In the current investigation, Cronbach’s α was 0.93 for the total score, intrusion = 0.89, avoidance = 0.80, and hyperarousal = 0.82.

For this study, we used a specific subscale of the CIES called ‘Current COVID-19 ED-effects’, which consists of 9 items rated on a 6-point Likert scale, to assess the impact of the COVID-19 pandemic social restrictions [42]. This questionnaire was developed and validated in different countries to evaluate specifically the effects of the pandemic on people with EDs, showing good reliability and good psychometric characteristics [43, 44]. The participants were asked to evaluate whether there was an effect (Not present = 0; Very increased = 5) of the social isolation compared to before February 2020 on nine specific domains: body concern, food restriction, weight change, binging, exercise, use of laxative, use of diuretics, purging, and body check. In the current investigation, Cronbach's α was 0.82.

### Statistical analysis

Due to the non-paired nature of our participant groups (anorexia nervosa patients and healthy siblings), we conducted independent statistical analyses. This approach considers the overall group differences while accommodating the variability within each group, aligning with the study’s design and objectives. To determine whether to include participants in the adolescent or adult group, we applied the World Health Organization’s definition, considering all individuals younger than 19 years old as adolescents. Differences in demographic characteristics were evaluated using t-tests for independent samples to evaluate the possible presence of differences between patients or HS enrolled in the two different Centers. The chi-square test was used to evaluate categorical data. A two-way MANOVA was conducted to assess the effect of ED diagnosis and age on psychological factors. Bonferroni correction was applied to control for multiple comparisons between subgroups to evaluate differences between subgroups, and partial eta square was evaluated as effect size for significant effects.

In the multiple regression analysis, an enter approach was employed, with a step-by-step method involving the construction of four distinct models. In each model, a different set of independent variables was entered to evaluate their effects on the IES-R global score. The first model included adolescent status (dichotomized as 1 for participants under 19 years old), the second model incorporated ED diagnosis (dichotomized as 1 for patients with a diagnosis), the third model examined the continuous variable SCL-58 scores using its relevant subscales, and the fourth model considered EDE-Q scores, also utilizing its respective subscales. This sequential approach enabled a comprehensive investigation of how each set of variables contributed to the IES-R global score.

All analyses were performed using IBM SPSS Statistics version 25.0. For all analyses, the alpha level was set to p < 0.05.

## Results

A total sample of 273 individuals was included in the study, of whom 154 were patients with an ED and 119 were HS. The clinical and psychological characteristics of the sample are reported in Table 1. Of the sample, 66 ED participants and 54 (45.4%) HS participants were adolescents. Looking at diagnoses, 55 patients fulfilled the criteria for anorexia nervosa, 51 people were diagnosed with bulimia nervosa, and 17 with binge eating disorder. Among them, there were 15 adolescents with anorexia nervosa (27.3%), 13 adolescents with bulimia nervosa (25.5%), and 3 with binge eating disorder (17.6%), with no differences for distribution (χ^2^ = 0.642, p = 0.725). No difference emerged between ED Centers as regards demographic (age: t = 0.935, p = 0.351; BMI: t = 1.526, p = 0.130) or psychological characteristics (EDE-Q total score: t = 0.833, p = 0.406; SCL58 global severity index: t = 0.299, p = 0.765). No difference emerged between people with ED with a healthy sibling and without (age: t = 0.348, p = 0.729; BMI: t = 1.675, p = 0.096; EDE-Q total score: t = 1.946, p = 0.084; SCL58 global severity index: t = 1.112, p = 0.256; IES-R total score: t = 0.938, p = 0.335).

**Table 1:** Demographic comparisons of the included sample

	aED N = 66	aHS N = 54	AED N = 88	AHS N = 65	F ED*Age	p pη^2^
Age	16.77 (1.41)	16.74 (1.45)	25.28 (7.46)	27.54 (8.90)	2.706	0.102
BMI	18.26 (2.88)	20.78 (2.32)	21.34 (6.79)	21.85 (3.69)	0.466	0.495
Years of education	8.35 (2.53)	8.69 (1.75)	13.33 (2.84)	14.01 (2.58)	0.910	0.341
Sex, F (%)	55 (83.33)	47 (87.04)	80 (90.91)	53 (81.54)	2.877*	0.090
Previous COVID-19 infection, yes (%)	5 (7.57)	2 (3.70)	7 (7.95)	7 (10.77)	2.299*	0.513
Current COVID-19 ED-effects	26.69 (8.32)	12.67 (9.13)	25.69 (8.49)	11.54 (9.02)	0.271	0.604
IES-R						
Intrusion	16.76 (7.91)	7.00 (7.04)	9.15 (6.20)	5.67 (4.90)	17.385	< 0.001
						0.154
Avoidance	15.33 (8.85)	5.97 (4.94)	8.77 (6.59)	4.84 (4.33)	9.085	0.003
						0.162
Hyperarousal	14.11 (7.09)	5.60 (4.90)	8.16 (5.44)	4.99 (4.63)	14.058	< 0.001
						0.160
IES-R total	46.20 (22.04)	18.57 (15.40)	26.08 (16.58)	15.49 (12.52)	15.901	< 0.001
						0.180
EDE-Q						
Restraint	3.56 (1.90)	0.41 (0.79)	2.79 (1.87)	0.59 (1.09)	4.500	0.035
						0.008
Eating concern	3.48 (1.42)	0.82 (1.47)	3.09 (1.66)	0.47 (0.94)	0.003	0.959
Shape concern	4.97 (1.25)	1.44 (1.79)	4.03 (1.82)	1.36 (1.44)	1.508	0.221
Weight concern	4.16 (1.64)	1.04 (1.44)	3.41 (2.03)	1.09 (1.25)	1.281	0.259
Global	4.04 (1.39)	0.93 (1.34)	3.33 (1.67)	0.88 (1.08)	1.106	0.294
SCL-58						
SOM	2.22 (1.07)	0.93 (0.92)	1.41 (0.84)	0.66 (0.58)	6.640	0.011
						0.125
OC	2.07 (1.11)	0.93 (0.96)	1.37 (0.95)	0.63 (0.65)	4.166	0.042
						0.084
IS	1.93 (1.17)	0.74 (0.79)	1.29 (0.86)	0.61 (0.63)	5.739	0.017
						0.187
D	2.12 (1.15)	0.93 (0.81)	1.53 (0.88)	0.72 (0.60)	3.485	0.065
A	2.17 (1.15)	1.11 (0.88)	1.55 (0.93)	0.80 (0.62)	2.354	0.127
GSI	2.15 (1.02)	0.94 (0.85)	1.50 (0.86)	0.71 (0.58)	4.496	0.035
						0.234

Means and standard deviations (SD) are reported in the table

*SOM* somatization, *OC* obsessive–compulsive, *IS* interpersonal sensitivity, *D* depression, *A* anxiety, *GSI* global severity index, *a* adolescent, *A* adult, *ED* eating disorder, *HS* healthy sibling

*Chi-square test

The main effect of age—but not looking at the ED condition—we found several significant differences. For all the comparisons, adolescents participants scored higher than adults showing a more severe psychological burden: intrusion (*F* = 15.364, *p* < 0.001 ), avoidance (*F* = 12.619, *p* < 0.001), hyperarousal (*F* = 10.548, *p* = 0.001), IES-R total score (*F* = 15.500, *p* < 0.001), EDE-Q shape (*F* = 5.221, *p* = 0.023), somatization (*F* = 15.202, *p* < 0.001), obsessive–compulsive (*F* = 10.757, *p* = 0.001), interpersonal sensitivity (*F* = 6.967, *p* = 0.009), depression (*F* = 8.743, *p* = 0.003), anxiety (*F* = 11.045, *p* = 0.001), and SCL-58 global severity index (*F* = 10.156, *p* = 0.002). The effect of the ED condition was significant for all the comparison, excluded age (*F* = 0.487, *p* = 0.486) and BMI (*F* = 3.392, *p* = 0.067): Current COVID-19 ED-effects (*F* = 115.496, *p* < 0.001), intrusion (*F* = 76.889, *p* < 0.001), avoidance (*F* = 52.784, *p* < 0.001), hyperarousal (*F* = 56.967, *p* < 0.001), IES-R total score (*F* = 75.297, *p* < 0.001), restrain (*F* = 104.709, *p* < 0.001), eating concern (*F* = 121.624, *p* < 0.001), shape concern (*F* = 135.364, *p* < 0.001), weight concern (*F* = 92.737, *p* < 0.001), EDE-Q total score (*F* = 137.521, *p* < 0.001), somatization (*F* = 84.039, *p* < 0.001), obsessive–compulsive (*F* = 59.527, *p* < 0.001), interpersonal sensitivity (*F* = 56.799, *p* < 0.001), depression (*F* = 60.769, *p* < 0.001), anxiety (*F* = 40.273, *p* < 0.001), and SCL58 global severity index (*F* = 72.029, *p* < 0.001).

Looking at the interaction between age and ED condition, we found that adolescents with ED presented the more severe condition, with significant effects for intrusion (*F* = 17.385, *p* < 0.001 ), avoidance (*F* = 9.085, *p* = 0.003), hyperarousal (F = 14.058, p < 0.001), IES-R total score (*F* = 15.901, *p* < 0.001), EDE-Q restrain (*F* = 4.500, *p* = 0.035), somatization (*F* = 6.640, *p* = 0.011), obsessive–compulsive (*F* = 4.166, *p* = 0.042), interpersonal sensitivity (*F* = 5.739, *p* = 0.017), and SCL-58 global severity index (*F* = 4.496, *p* = 0.035).

**Fig. 1:**
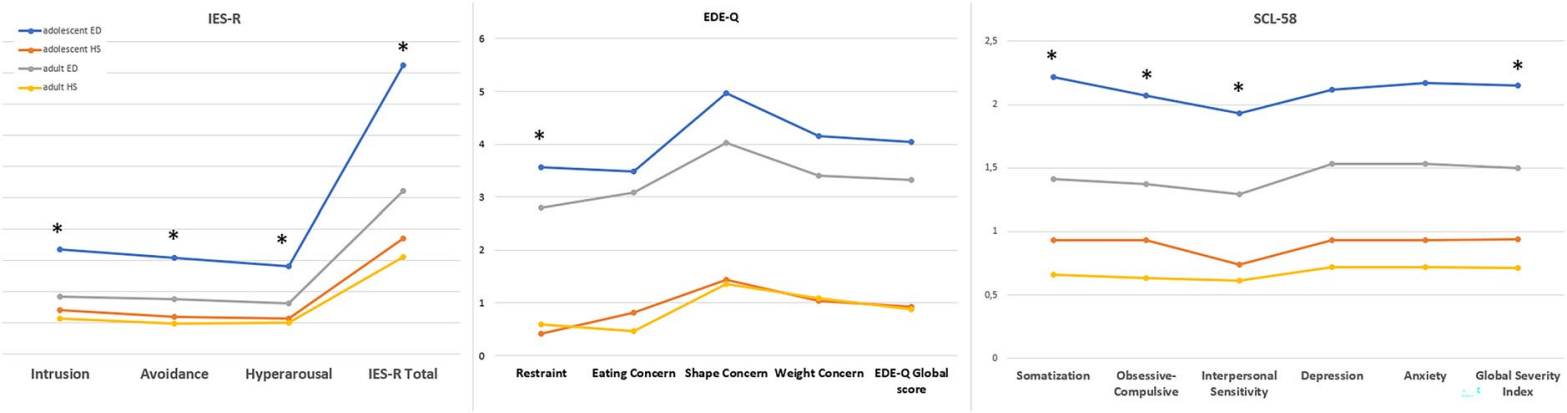
Graphical representation of the scores divided by age group. The scores indicate that the adolescent patients’ subgroup is the one that reported the highest scores on all the subscales. Moreover, it is the only group that passed the clinical cutoff for significant posttraumatic symptomatology. *Indicates significant interaction ED*age

See Table 2 for pairwise comparisons between subgroups and Fig. 1 for a graphical representation of the questionnaire results. Adolescents with EDs exhibited a higher psychological burden, whereas adult HSs had lower scores. Intrusion was found to be the only feature that showed a significant difference between adolescent and adult HSs, with adolescents scoring higher.

**Table 2:** Pairwise comparisons between ED and HS adolescents and adults for the ED*Age effect

	Post hoc analyses
IES-R	
Intrusion	aED > aHS (*p* < 0.001) aED > AED (*p* < 0.001) aED > AHS (*p* < 0.001) AED > aHS (*p =* 0.035) aHS > AHS (*p =* 0.003)
Avoidance	aED > aHS (*p* < 0.001) aED > AED (*p* < 0.001) aED > AHS (*p* < 0.001) AED > AHS (*p =* 0.001)
Hyperarousal	aED > aHS (*p* < 0.001) aED > AED (*p* < 0.001) aED > AHS (*p* < 0.001) AED > aHS (*p =* 0.025) AED > AHS (*p =* 0.004)
IES-R total	aED > aHS (*p* < 0.001) aED > AED (*p* < 0.001) aED > AHS (*p* < 0.001) AED > aHS (*p =* 0.020) AED > AHS (*p =* 0.001)
EDE-Q	
Restraint	aED > aHS (*p* < 0.001) aED > AED (*p =* 0.030) aED > AHS (*p* < 0.001) AED > aHS (*p* < 0.001) AED > AHS (*p* < 0.001)
SCL-58	
Somatization	aED > aHS (*p* < 0.001) aED > AED (*p* < 0.001) aED > AHS (*p* < 0.001) AED > aHS (*p =* 0.014) AED > AHS (*p* < 0.001)
Obsessive–compulsive	aED > aHS (*p* < 0.001) aED > AED (*p =* 0.042) aED > AHS (*p* < 0.001) AED > aHS (*p* < 0.001) AED > AHS (*p* < 0.001)
Interpersonal sensitivity	aED > aHS (*p* < 0.001) aED > AED (*p* < 0.001) aED > AHS (*p* < 0.001) AED > aHS (*p =* 0.008) AED > AHS (*p* < 0.001)
Global Severity Index	aED > aHS (*p* < 0.001) aED > AED (*p* < 0.001) aED > AHS (*p* < 0.001) AED > aHS (*p =* 0.003) AED > AHS (*p* < 0.001)

The table reported pairwise comparisons with Bonferroni correction. For means, standard deviations, F and p-values see Table 1

*a* adolescent, *A* adult, *ED* eating disorder, *HS* healthy sibling

From the multiple regression analysis, it was observed that being an adolescent (B = 0.151, 95% CI = 2.345–10.338), depression (B = 0.313, 95% CI = 0.354–12.734), and eating restraint (B = 0.244, 95% CI = 0.850–3.991) played a role in determining distress levels assessed after the pandemic. The presence of an ED diagnosis was significant in the models without EDE-Q subscales (Model 3, B = 0.130, 95% CI = 1.069–9.676). The details of the evaluated models are presented in Table 3.

**Table 3:** Multiple regression analysis with IES-R total score as dependent variable

	β	SE	t	p
Model 1: F = 27.737, p < 0.001, R^2^ = 0.109				
Adolescent	13.90	2.64	5.27	< 0.001
Model 2: F = 44.339, p < 0.001, R^2^ = 0.283, DR^2^ = 0.173				
Adolescent	12.02	2.39	5.03	< 0.001
ED	17.25	2.34	7.37	< 0.001
Model 3: F = 37.580, p < 0.001, R^2^ = 0.545, DR^2^ = 0.262				
Adolescent	6.25	2.04	3.06	0.002
ED	5.37	2.18	2.46	0.015
Somatization	5.94	3.38	1.75	0.081
Obsessive–compulsive	− 0.88	2.73	− 0.32	0.746
Interpersonal sensitivity	3.93	2.86	1.37	0.171
Depression	6.79	3.08	2.21	0.028
Anxiety	− 3.33	2.62	− 1.27	0.205
Model 4: F = 26.036, p < 0.001, R^2^ = 0.570, DR^2^ = 0.025				
Adolescent	6.34	2.03	3.13	0.002
ED	2.45	2.66	0.92	0.358
Somatization	3.69	3.41	1.08	0.281
Obsessive–compulsive	− 0.44	2.69	− 0.16	0.869
Interpersonal sensitivity	3.24	2.83	1.15	0.253
Depression	6.36	3.05	2.09	0.038
Anxiety	− 2.60	2.60	− 0.99	0.319
Restraint	2.42	0.80	3.04	0.003
Eating concern	− 1.50	1.29	− 1.16	0.246
Shape concern	0.63	1.44	0.44	0.661
Weight concern	0.41	1.41	0.29	0.773

*Β* unstandardized coefficient, *SE* standard error

## Discussion

This study was designed to investigate the impact of the COVID-19 pandemic on people with ED and their unaffected siblings, looking at the possible effects of age. The findings of our research shed light on the differential effects of the pandemic on various age groups, revealing that adolescent patients with EDs faced more substantial challenges than other subgroups.

Remarkably, adolescents with EDs exhibited significantly higher scores for clinical posttraumatic symptomatology across all evaluated scales. This suggests that the COVID-19 pandemic has constituted a significant stressor for young individuals, especially those grappling with EDs [45]. This observation aligns with global trends in the ED literature, which has reported a surge in hospitalizations for ED symptomatology post-lockdown, particularly among young people [6]. However, it's worth noting that there has also been a surge in help-seeking for other psychiatric conditions among youth, including obsessive–compulsive disorders, anxiety disorders, and emotional reactivity [46–48].

Posttraumatic symptomatology holds particular importance within the realm of individuals with EDs [49–51], although not all data fully corroborate this. For instance, some patients reported experiencing relief due to reduced social pressures associated with decreased comparisons, controls, and exposure [52, 53]. However, this positive effect was not observed among adolescents [54], who may inhabit stressful environments marked by familial conflicts related to EDs. The existing literature underscores the diverse burdens carried by patients, siblings, and caregivers, emphasizing the significance of understanding the pandemic’s impact on mental health trajectories [26, 55, 56], and calling for studies on these populations. This highlights the need for further studies within these populations, offering valuable insights for enhancing prevention strategies and service provisions, including a shift towards telehealth and online support, a greater focus on mental health, improved access to care, more personalized treatment approaches, and a rise in community support initiatives [26, 28].

Additionally, routine habits and daily activities may serve as coping strategies to mitigate uncertainty and the challenges posed by social distancing, factors that have contributed to worsening youth psychopathology [44, 57]. Individuals with EDs tend to exhibit reduced coping strategies in response to advertising environments [58, 59], and these limitations may become particularly evident in contexts like the pandemic. Nevertheless, longitudinal studies are essential to assess the long-term effects of these factors.

Adolescence emerged as a stronger predictor of psychological distress, with age-related elements like concerns about life trajectories and exposure to information overload potentially contributing to this result. These distinctions between adolescents and adults appear to be substantiated in the existing body of literature, which indicates a higher prevalence of depression and anxiety in young adults. This can be comprehended within the framework of the stress and coping model, which proposes that individuals assess the significance of stressors (primary appraisal) and evaluate their personal perceived resources (e.g., coping strategies) for managing emotions or addressing the given stressor [60, 61]. Furthermore, the cumulative life experiences of older adults may bolster their resilience in dealing with analogous situations [62]. Interestingly, among the various constructs evaluated, the only EDE-Q subscale that significantly emerged was restraint eating, as predictor of COVID-related stressor. This psychopathological construct has previously been identified as a core feature among adolescent patients, with evidence pointing to neurobiological adaptive mechanisms related to inhibition processes [63] and its central role in ED psychopathology [64]. Moreover, restrictive thoughts have been highlighted as a robust bridge between EDs and posttraumatic psychopathologies, showing the possible effects that dysfunctional behaviors might have on modulation of symptomatology [65]. On the other hand, intrusive thoughts were the only construct that emerged significantly higher among adolescent unaffected siblings compared to their adult counterparts. The presence of intrusive thoughts following the COVID-19 pandemic has been noted in adolescents [66] and has been associated with direct and indirect exposure to stressful events. This could be a vulnerability factor in youth contributing to psychological distress, especially among ED patients [67–69].

In conclusion, the current body of literature underscores the pressing need for tailored interventions to mitigate the exacerbation of health, social, and academic disparities laid bare by the COVID-19 pandemic [70, 71]. This imperative is particularly pronounced when considering adolescents. Once again, our data accentuate the crucial importance of directing attention to the specific needs of youth in the ED field, encompassing their cognitive, emotional, and social requirements [72, 73].

### Strengths and limitations

This study boasts several strengths that stem from its recruitment process, which involved participants and their siblings from two specialized centers. This approach provided a robust foundation for clinical evaluation, enhancing the reliability and validity of our findings.

Nonetheless, it is important to acknowledge certain limitations inherent in our study. Firstly, our reliance on self-reported questionnaires may introduce the potential for participants to misreport their cognitive and emotional states, possibly impacting the accuracy of the data collected. Secondly, the absence of data regarding the duration of EDs in most participants restricted our ability to explore the potential role of the disorders’ duration in influencing outcomes. Thirdly, we didn’t evaluate the presence of specific comorbidities in patients and this element could have an effect on results. Lastly, it’s essential to recognize that this study follows a cross-sectional design, which limits our ability to draw causal inferences. While our findings highlight associations and trends, they do not establish causation. Longitudinal research could provide a more comprehensive understanding of the dynamics between the variables studied and replication of our results is needed to corroborate these findings, even with larger samples.

## Conclusion

In summary, this study has underscored the distinctive impact of the COVID-19 pandemic on the mental health of adolescents with eating disorders (EDs), reaffirming their specific vulnerability in the pandemic’s landscape. Adolescents, as a high-risk group for maladaptive psychological functioning, should be at the forefront of future mental health planning. Tailored support for adolescents with EDs is imperative. Following the lockdowns imposed during the pandemic, mental healthcare services must now prioritize the development of supportive interventions. These interventions should aim to provide a nurturing environment for psychological growth, even in the face of ongoing challenges such as social distancing measures and disruptions to daily routines. In conclusion, our findings emphasize the urgency of recognizing and addressing the unique needs of adolescents with EDs in the aftermath of the pandemic. By doing so, we can better equip this vulnerable group to navigate the complex terrain of mental health during and beyond times of crisis.

## Data Availability

The datasets used and analyzed during the current study are available from the corresponding author on reasonable request.
